# The effects of backpack carriage on gait kinematics and kinetics of schoolchildren

**DOI:** 10.1038/s41598-019-40076-w

**Published:** 2019-03-04

**Authors:** Habibah N. Ahmad, Tiago M. Barbosa

**Affiliations:** Physical Education and Sports Science Academic Group, National Institute of Education, Nanyang Technological University, Singapore, Singapore

## Abstract

There has been a growing concern among clinical and educational practitioners, as well as, policy makers on the use of backpacks by schoolchildren. On a daily basis, pupils spend a significant amount of time carrying stuffed and heavy backpacks. The aim of this study was to investigate the effects of backpack carriage with different loads on spatiotemporal parameters of gait, plantar pressure and force distribution under different foot regions in schoolchildren. We have assessed fifty-seven primary school students (7–9 years-old) performing four walks of 10 m (carrying 0%, 10%, 15% of body mass in the backpack and the load they brought to school). A floor-based photocell system was used to collect the gait kinematics and insoles capacitive pressure sensors the kinetics. Children walked slower and at lower cadence with the load brought to school than in the other three conditions. There was no significant main effect on stride length. Backpack carriage with different loads did have a significant effect on plantar pressure and force distribution. We noted that heavier the load, higher the pressure and force under different foot regions. Our findings highlight that gait biomechanics of children (such as stride kinematics and pressure under the feet) is affected by carrying loads in the backpacks.

## Introduction

Children and adolescents, over their primary and secondary school years, have the daily routine of carrying school materials. School materials are commonly put inside a backpack because it is deemed as a convenient way to carry loads^1,2^. Hence, on a daily basis, pupils spend a significant amount of time, carrying stuffed and heavy backpacks.

Studies have noted that load carriage may alter gait kinematics^3–5^, ground reaction force^6^ and plantar pressure^2^. The biomechanical changes in static or dynamic posture caused by load carriage can contribute to musculoskeletal injury^7^. For instance, back pain^1^, joint problems^4^ and foot blisters^8^. Nevertheless, a systematic review reported that there is no consistent pattern of association between schoolbag use or type and back pain^9^. Upon reviewing 5 prospective longitudinal studies and 64 cross-sectional or retrospective studies (total n = 72,627) the authors concluded that schoolbag characteristics (weight, design and carriage method) do not increase the risk of developing back pain in children. Nevertheless, it is unclear what other biomechanical and motor control changes might occur in the gait. Thus, due to the possible changes caused by load carriage in gait biomechanics and motor control strategies, there has been a growing concern among clinical and educational practitioners, as well as, policy makers on the use of backpacks by schoolchildren. Arguably, one may claim that this concern seems to be more evident in countries that place much emphasis on the wellbeing of children, such as those topping international education rankings.

Previous studies reported that heavier load conditions did not result in a significant change in spatiotemporal parameters such as stride length, cadence, duration of the swing and double support phases^10,11^. However, others reported that as the backpack load increased in weight, there was likewise a significant increase in the double support and a decrease in swing durations^5,12,13^. Therefore, it remains unclear if there is a change or not in the gait kinematics of schoolchildren carrying loads on the back.

Two studies reported the plantar pressure of children carrying a loaded backpack but while standing still^11,14^. Most studies on the gait kinetics carrying loads in a backpack reported the changes in ground reaction force^6^. One study assessed the plantar pressure distribution, but in adults^2^. Literature reports mixed findings, as some papers shared an increase in the ground reaction force or plantar pressure with increasing loads;^2,6^ whereas, at least one failed to note significant differences^14^. One single research reported the plantar pressure of schoolchildren walking but for a short distance (4 and 8 m), at a laboratory, and comparing children in only two conditions (carrying the load brought to school and unloaded)^11^. It is also still unclear the hypothetical changes in gait biomechanics with increasing loads. Thus, one can claim that a research design should take these gaps in the literature and limitations into consideration. At such early age, being assessed in an unfamiliar and, for some children, intimidating venue as a laboratory can often trigger the “Hawthorne effect”. To tackle such concerns, the advice would be to collect the data at school settings and selecting a setup that does not impose significant gait constraints. Nevertheless, we have failed to find a research taking into consideration the ecological validity of the data collected.

The aim of this study was to investigate the effects of backpack carriage with different loads on the gait kinematics and kinetics in schoolchildren. It was hypothesised that with increasing load, there is as well an increase in the mechanical load and a change in the spatiotemporal parameters of the gait.

## Results

### Kinematics

There was no load x school level interaction in all selected variables (Table 1). No main effect was found for school level in most variables, except the duration of the swing phase.

**Table 1:** Descriptive and inferential statistics of the gait kinematics at different loading conditions in the backpack.

Variable	SL	Initial load mean ± 1 SD	0% BM mean ± 1 SD	10% BM mean ± 1 SD	15% BM mean ± 1 SD	SL x load interaction	SL main effect	Load main effect
Velocity [m/s]	P1	1.09 ± 0.16	1.18 ± 0.20	1.24 ± 0.21	1.19 ± 0.18	F = 0.87	F = 2.03	F = 10.78
	P2	0.99 ± 0.11	1.10 ± 0.13	1.11 ± 0.12	1.09 ± 0.11	p = 0.52	p = 0.14	p < 0.001
	P3	1.11 ± 0.26	1.16 ± 0.22	1.16 ± 0.20	1.14 ± 0.20	ƞ² = 0.50	ƞ² = 0.07	ƞ² = 0.38
Cadence [step/min]	P1	120.86 ± 13.03	126.74 ± 14.16	133.04 ± 18.57	128.61 ± 13.01	F = 0.71	F = 3.28	F = 8.40
	P2	116.04 ± 13.46	120.10 ± 12.89	126.41 ± 11.01	123.76 ± 11.18	p = 0.63	p = 0.05	p < 0.001
	P3	113.87 ± 14.29	119.10 ± 13.10	120.16 ± 14.78	117.26 ± 15.02	ƞ² = 0.04	ƞ² = 0.11	ƞ² = 0.33
Stride length [m]	P1	1.08 ± 0.08	1.11 ± 0.13	1.12 ± 0.09	1.11 ± 0.11	F = 1.23	F = 3.28	F = 0.96
	P2	1.03 ± 0.11	1.10 ± 0.12	1.05 ± 0.07	1.06 ± 0.09	p = 0.30	p = 0.05	P = 0.42
	P3	1.17 ± 0.19	1.17 ± 0.15	1.16 ± 0.12	1.16 ± 0.10	ƞ² = 0.65	ƞ² = 0.11	ƞ² = 0.52
Stance phase [s]	P1	0.63 ± 0.07	0.60 ± 0.08	0.57 ± 0.07	0.58 ± 0.06	F = 12.58	F = 2.90	F = 0.81
	P2	0.65 ± 0.07	0.60 ± 0.06	0.60 ± 0.06	0.60 ± 0.07	p = 0.56	p = 0.06	p < 0.001
	P3	0.66 ± 0.09	0.62 ± 0.07	0.62 0.07	0.64 ± 0.09	ƞ² = 0.04	ƞ² = 0.98	ƞ² = 0.42
Swing phase [s]	P1	0.39 ± 0.04	0.38 ± 0.04	0.36 ± 0.04	0.37 ± 0.04	F = 0.96	F = 5.85	F = 7.04
	P2	0.40 ± 0.05	0.38 ± 0.03	0.38 ± 0.03	0.37 ± 0.03	p = 0.46	p = 0.01	p < 0.001
	P3	0.42 ± 0.05	0.41 ± 0.04	0.40 ± 0.04	0.41 ± 0.05	ƞ² = 0.05	ƞ² = 0.18	ƞ² = 0.29
Double support phase [s]	P1	0.23 ± 0.04	0.20 ± 0.06	0.20 ± 0.04	0.21 ± 0.04	F = 8.63	F = 0.50	F = 13.90
	P2	0.25 ± 0.06	0.21 ± 0.04	0.21 ± 0.05	0.23 ± 0.06	p = 0.53	p = 0.61	p < 0.001
	P3	0.22 ± 0.06	0.21 ± 0.04	0.21 ± 0.05	0.22 ± 0.06	ƞ² = 0.05	ƞ² = 0.02	ƞ² = 0.44

We found significant and moderate main effects of the load on the velocity (F = 10.78; p < 0.001; ƞ² = 0.38), cadence (F = 8.40; p < 0.001; ƞ² = 0.33), durations of the stance (F = 0.81; p < 0.001; ƞ² = 0.42), swing (F = 7.04; p < 0.001; ƞ² = 0.29) and double support phases (F = 13.90; p < 0.001; ƞ² = 0.44). Children walked slower (p < 0.001; 0.44 < d < 0.56) and at lower cadence (p ≤ 0.001; 0.45 < d < 0.65) with the load brought to school than in the remaining conditions. As such, the stance (p < 0.001; 0.57 < d < 0.71), swing (p < 0.001; d = 0.44) and double support (p < 0.001; d = 0.40) phases took longer. There was no significant main effect on the stride length.

### Kinetics

We noted significant and strong load x school level interactions in the mean pressure (33.704 < F < 275.64; p < 0.001; 0.19 < ƞ² < 0.91) (Tables 2 and 3). Mean pressure increased with school level and the amount of load to be carried. No significant and trivial load x school level interactions were noted in the remaining variables. Likewise, the main trend was for no significant and trivial main effects of the school level in these variables, except the pressure-time integral under the heels (F = 4.881; p = 0.01; ƞ² = 0.15).

**Table 2:** Descriptive statistics of the gait kinetics at different loading conditions in the backpack.

Variable	SL	Initial load mean ± 1 SD				0% BM mean ± 1 SD				10% BM mean ± 1 SD				15% BM mean ± 1 SD			
		Toes	Mtt	Mid	Heels	Toes	Mtt	Mid	Heels	Toes	Mtt	Mid	Heels	Toes	Mtt	Mid	Heels
Mean Pressure [kPa]	P1	72.85 ±17.43	63.14 ±11.04	33.82 ±5.55	83.17 ±12.37	65.59 ±14.18	58.44 ±11.12	32.36 ±5.85	81.97 ±10.75	67.56 ±16.04	60.73 ±10.69	33.74 ±6.47	88.49 ±13.57	69.57 ±18.07	62.39 ±16.63	35.41 ±6.82	86.55 ±13.10
	P2	73.34 ±12.88	64.33 ±8.12	33.13 ±6.60	82.63 ±11.81	71.25 ±14.95	59.92 ±7.47	32.03 ±5.70	77.06 ±14.98	74.34 ±15.34	62.62 ±7.64	32.89 ±6.99	83.09 ±6.99	73.18 ±14.48	65.01 ±8.31	35.24 ±8.76	83.65 ±13.93
	P3	73.96 ±15.97	71.85 ±13.68	35.23 ±9.63	96.40 ±23.97	77.41 ±17.08	64.08 ±12.98	34.24 ±10.81	92.04 ±21.86	79.97 ±21.88	66.09 ±14.83	35.61 ±10.24	94.22 ±22.82	118.11 ±31.37	197.62 ±43.60	155.32 ±41.96	279.42 ±60.44
Contact area [cm^2^]	P1	13.59 ±0.34	29.03 ±0.69	37.56 ±3.81	29.03 ±0.75	13.65 ±0.27	29.02 ±0.79	36.47 ±3.90	28.73 ±0.95	13.61 ±0.31	28.92 ±0.81	36.85 ±4.06	28.87 ±0.74	13.59 ±0.34	28.88 ±0.82	37.69 ±3.27	28.75 ±0.94
	P2	13.66± 0.37	29.13 ±0.60	37.61 ±3.81	28.69 ±1.20	13.62 ±0.41	28.90 ±1.04	36.95 ±3.85	28.40 ±1.18	13.62 ±0.41	29.07 ±0.69	36.69 ±3.25	28.38 ±1.53	13.66 ±0.37	29.13 ±0.60	37.46 ±3.63	28.42 ±1.41
	P3	13.71± 0.18	29.15 ±0.67	36.95 ±5.22	29.16 ±0.91	13.71 ±0.18	28.96 ±0.80	35.12 ±5.15	28.85 ±1.20	13.75 ±0.25	28.76 ±1.01	35.41 ±5.07	28.47 ±1.48	13.75 ±0.33	28.94 ±0.60	35.85 ±4.88	28.57 ±1.45
Pressure-time integral [kPa.s]	P1	122.45± 52.71	175.52 ±61.69	174.91 ±36.42	241.06 ±56.76	97.10 ±35.97	152.27 ±37.17	168.46 ±39.10	243.21 ±55.52	87.73 ±28.22	143.43 ±49.49	161.68 ±31.14	248.26 ±61.99	102.34 ±44.98	156.24 ±49.18	170.42 ±36.82	264.90 ±87.96
	P2	130.67± 53.67	195.31 ±43.78	173.69 ±30.05	214.81 ±38.70	109.08 ±40.54	173.48 ±39.39	157.05 ±25.98	198.90 ±41.64	113.46 ±40.01	167.90 ±42.04	152.83 ±35.36	193.59 ±42.91	108.82 ±38.35	108.82 ±38.35	154.39 ±36.46	165.55 ±43.86
	P3	128.34± 67.34	179.60 ±59.14	147.77 ±50.89	206.88 ±67.75	115.19 ±44.30	168.63 ±56.77	147.41 ±48.65	203.28 ±66.66	120.17 ±44.83	162.12 ±53.65	145.51 ±47.02	202.95 ±70.04	138.35 ±56.09	138.35 ±56.09	149.90 ±50.60	208.15 ±76.23
Force-time integral [N.s]	P1	78.88± 31.84	211.31 ±77.76	207.92 ±50.84	276.12 ±61.75	62.31 ±20.58	179.25 ±46.85	187.56 ±49.09	265.16 ±53.82	58.02 ±17.97	170.77 ±63.26	182.44 ±50.99	268.77 ±48.82	64.89 ±24.30	189.83 ±70.59	188.97 ±51.40	274.02 ±69.01
	P2	85.39 ±35.01	246.88 ±64.13	207.53 ±62.87	261.87 ±64.05	72.81 ±27.59	207.56 ±51.30	178.07 ±48.45	237.52 ±59.67	73.18 ±25.76	205.75 ±49.08	171.62 ±57.94	227.81 ±63.78	69.38 ±28.65	204.13 ±64.45	170.18 ±69.09	221.65 ±76.55
	P3	76.92 ±39.15	225.44 ±92.95	174.88 ±72.91	270.09 ±107.1	74.32 ±31.25	199.03 ±76.20	166.21 ±65.35	256.61 ±109.5	75.59 ±31.18	196.06 ±65.02	170.41 ±65.65	253.53 ±106.2	87.30 ±40.74	206.81 ±74.46	176.84 ±81.97	262.93 ±126.3

SL – school level, Mtt – metatarsus, Mid – midfoot, 0% BM – no backpack, 10% BM – 10% of body mass in load inside the backpack, 15% BM - 15% of body mass in load inside the backpack.

**Table 3:** Inferential statistics of the gait kinetics at different loading conditions in the backpack. SL – school level, Mtt – metatarsus, Mid – midfoot.

Variable	Statistics	Toes			Mtt			Mid			Heel		
		F	p	η^2^	F	p	η^2^	F	p	η^2^	F	p	η^2^
Mean Pressure	Load x SL inter.	33.704	<0.001	0.39	231.41	<0.001	0.90	217.18	<0.001	0.89	275.64	<0.001	0.91
	Load main effect	34.735	<0.001	0.56	251.39	<0.001	0.82	237.53	<0.001	0.82	290.21	<0.001	0.84
	SL main effect	6.294	<0.001	0.19	42.659	<0.001	0.61	44.600	<0.001	0.62	46.038	<0.001	0.63
Contact area	Load x SL inter.	0.884	0.50	0.03	1.001	0.43	0.01	1.414	0.21	0.05	0.882	0.50	0.03
	Load main effect	0.473	0.70	0.01	2.007	0.10	0.04	15.600	<0.01	0.22	4.659	0.01	0.08
	SL main effect	2.835	0.07	0.10	0.456	0.64	0.02	0.255	0.78	0.01	0.568	0.58	0.02
Pressure-time integral	Load x SL inter.	2.257	0.06	0.08	1.215	0.31	0.04	1.045	0.40	0.04	1.561	0.16	0.05
	Load main effect	7.189	<0.001	0.12	11.593	<0.001	0.18	3.191	0.04	0.06	0.917	0.44	0.02
	SL main effect	0.992	0.38	0.04	1.098	0.34	0.04	1.481	0.24	0.05	4.881	0.01	0.15
Force-time integral	Load x SL inter.	3.285	0.02	0.11	0.691	0.66	0.03	4.212	0.30	0.04	0.780	0.56	0.03
	Load main effect	6.276	0.01	0.10	15.820	<0.001	0.23	4.803	0.01	0.08	2.284	0.09	0.04
	SL main effect	0.990	0.38	0.04	0.935	0.40	0.03	0.583	0.56	0.02	0.944	0.39	0.04

The load had a main effect on the contact area under the midfoot and heel (4.659 < F < 15.600; 0.001 < p < 0.01; 0.08 < ƞ² < 0.22). There was a significant and moderate-strong main effect of the load on the selected pressure-time and force-time integrals (3.191 < F < 15.820; 0.001 < p < 0.04; 0.06 < ƞ² < 0.23). Larger contact area under midfoot and heel was observed when students carried the load brought to school as compared to the other three conditions (0.001 < p < 0.04; 0.17 < d < 0.35). Overall, pressure-time integral (0.001 < p < 0.03; 0.12 < d < 0.64) and force-time integral (0.001 < p < 0.03; 0.11 < d < 0.72) under the toes, metatarsus and, midfoot increased moderate-strongly with heavier loads.

## Discussion

The aim of this study was to investigate the effects of backpack carriage with different loads on spatiotemporal parameters of gait, plantar pressure and force distribution under different foot regions in schoolchildren. We noted that children walked slower and at lower cadence with the load brought to school than in the remaining conditions, but no change in stride length. Mean pressure increased with school level and the amount of load carried. Pressure-time and force-time integrals under the feet also increased with heavier loads carried.

On average, children brought to school 15% of their body mass as load inside their backpacks. Younger students (6–8 years-old) were almost five times more likely to carry heavier backpacks than older students (9–14 years-old)^15^. It was recommended that schoolchildren should carry up to 15% of their body mass^16^. Referring to the 95CI (P1: 13.68–17.31%; P2: 13.30–16.89%; P3: 12.29–15.84%) one can learn that overall the vast majority of the students adhere to this guideline. The mean and 95CI values are within^15–17^ or less^10,18^ than what is reported in the literature.

There was neither load x school level interaction nor a school level main effect. As such, the school level, and, hence the chronological age, seems not to have a key-role. Children walked slower, at lower cadence with the load brought to school than in the remaining conditions. Therefore, the stance, swing and double support phases took longer.

However, comparing 0%BM, 10%BM and 15%BM there were trivial and non-significant variations in spatiotemporal variables. This is in tandem to what was reported previously^10,13^. The lack of change in the spatiotemporal parameters of the gait pattern with increasing loads points out to hypothetical adaptations at other levels, such as physiological and kinetic responses. Others reported that despite no change noted in spatiotemporal parameters of the gait, the acute physiological response was prone to increase, such as cardiovascular and respiratory adaptations^10,19^. For instance, energy expenditure increased with load, notably from 15%BM onwards^20^. Lower-limbs electromyographic adaptations have also been reported with increasing loads, at least in adults^21^.

Altogether, we noted that children have a different spatiotemporal pattern while walking with the load that they are used to carrying on a regular basis. A change in that load yielded significant changes in the gait kinematics. This could be due to the children adapting to a new set of interacting motor control constraints (i.e., task, environment, organismic)^22^. Comparing the three imposed load constraints (i.e., 0%BM, 10%BM and 15%BM) participants self-organised the control of their locomotion system to deliver similar gait kinematics. Indeed, motor performance theories emphasize the importance of self-organization and autonomous properties as an example of the dynamical behaviour of complex nonlinear systems, such as human locomotion^23^.

Significant and strong load x school level interactions were noted by us in the mean pressure under the four regions of the feet. Mean pressure increased with school level and the amount of load carried. As in other researches, mean pressure increased with heavier loads^11^. Interestingly, the changes in plantar pressure were stronger when standing still than walking^11^. Increased moments and power at the hip, knee and ankle showed increasing demand with backpack load^24^. Such increase in the mechanical load will be reflected on the plantar pressure as noted in this research. Mean pressure was highly correlated with peak pressure (r = 0.90 ± 0.09)^25^. As such, changes reported for mean pressure are expected to be the same in the case of the peak pressure. It is also noteworthy that this change was also affected by school level, where mean pressure increased for schoolchildren from Primary 1 to Primary 3. Literature is scarce on this matter as in most cases authors did not provide a breakdown of the data by chronological age or school level. Therefore, follow-up research should be conducted to understand the changes over primary and/or secondary school levels.

Contact area under midfoot and heel was larger when students carried the load brought to school than the three other conditions. This may suggest a change in the foot strike pattern, where participants shifted slightly the pattern carrying loads that they were not used to. While carrying the habitual load, students selected a heel strike pattern; conversely, they shifted to more of a midfoot strike pattern in the three other trials. Concurrently, an increase in the load imposed an increase in the area under the midfoot. Past studies also reported that modifications appear to happen mostly in the midfoot, with an increase in contact area carrying heavier loads^11,26^. Nevertheless, contact areas increase less markedly when walking than standing^11^.

Pressure-time and force-time integrals provided insights on the changes of the kinetics over the stance phase. Because there was no change in the duration of the stance phase when the load was fixed (0%BM, 10%BM and 15%BM), but with mean pressure increased, both parameters increased moderate-strongly with heavier loads under the toes, metatarsus and midfoot. This can be due to the changes in the foot strike pattern aforementioned, when children hypothetically shifted from heel to midfoot strike pattern. Nevertheless, variables most commonly used to characterise plantar pressures (peak pressure, mean pressure and pressure-time integrals) are highly inter-correlated^25^. So, findings regarding pressure-time and force-time integrals are consistent with what we discussed on mean pressure.

Overall, walking with an unusual amount of load led to a change in the foot strike pattern, which in turn changed the gait kinematics and kinetics. Increasing the fixed load to be carried, there was likewise an increase in the plantar pressure and force distribution. Children’s perception of school backpack weight has been explored in the past. Children have been shown to be poor judges of schoolbag weight^27^. Children who perceived their bag to be heavy were also more likely to report pain or discomfort^28^. It is unclear if psychological factors as this one played a role in the outcomes of our research. That said, pupils’ perception on the weight of the backpack should be taken into consideration by parents, teachers, school leaders and researchers.

It can be addressed as main limitations of our study: (i) one should refrain from inferring any causality between changes in gait biomechanics reported in this study and acute or chronic musculoskeletal injuries, syndromes or conditions; (ii) a deeper insight on gait changes could be gathered selecting other setups and experimental techniques, such as motion-capture systems and force plates. However, this could reduce the ecological validity of the research; (iii) one should refrain from extrapolating these findings to other cohorts of students, such as those in upper-primary or secondary school levels and lastly; (iv) the way backpacks were worn was standardised to be symmetrical, although some participants might prefer other carriage methods.

In conclusion, we found a change in temporal parameters of the gait while carrying unusual loads (the load brought to school vs. the three other conditions). Pressure and force distribution increased with load and school level. As such, children carrying loads in the backpack had significant changes in their gait biomechanics.

## Methods

### Participants

We recruited sixty primary school students aged between 7 and 9 years-old to take part in this research. Students were recruited by advertisement in schools administrated by the Ministry of Education. Of the 60 students, 21 were in Primary 1 (7 years-old), 19 in Primary 2 (8 years-old) and 20 in Primary 3 (9 years-old). Three students were unable to finish their participation and as such, the final sample size was 57 (Table 4).

**Table 4:** Participants’ anthropometrics and backpack loads (mean ± 1 SD).

School level	N	Age [years-old]	Height [m]	Body mass [kg]	Load to body mass brought to school [%]
P1	18	7	1.27 ± 0.04	24.77 ± 4.14	15.50 ± 3.65
P2	19	8	1.31 ± 0.05	27.69 ± 4.55	15.10 ± 3.72
P3	20	9	1.37 ± 0.06	32.23 ± 7.95	14.07 ± 3.80

Inclusion criteria included: (i) participants (boys or girls) must carry a backpack to school on a daily basis (i.e. trolley or other kinds of bag were not deemed as suitable); (ii) aged between 7 and 9 years-old; (iii) enrolled in either Primary 1, 2 or 3; (iii) no musculoskeletal injury in the past 6 months; (iv) no clinical diagnose of musculoskeletal or neurological diseases, syndrome or condition.

The Institutional Review Board of Nanyang Technological University approved all experimental procedures. All methods were performed in accordance with the relevant guidelines and regulations. Permission to collect data in primary schools was approved as well by the Ministry of Education (Singapore). All children and parents or guardians were furnished with the necessary verbal and written information. Written informed consent was obtained from parents and/or legal guardian and children.

### Procedures

A randomised crossover design was selected for this research. The study took place at the participants’ primary schools. Mass of the backpack, body mass and height were measured with a weighing scale and stadiometer, respectively (Seca, Hamburg, Germany).

After familiarisation with apparatus and procedures, children were invited to undergo four walks of 10 m on a levelled, consistent surface, at self-selected pace: (i) with the backpack weight they brought to school; (ii) without backpack (i.e., unloaded, with 0% of body mass); (iii) with 10% of their body mass (school material such as textbooks and notebooks) inside the backpack and; (iv) with 15% of their body mass in the backpack. Epidemiological, physiological and biomechanical data suggested a load threshold of 10–15% body mass^16^. The participants, in school attire, held the backpack by both straps fully adjusted over their shoulders (one strap on each shoulder). Table 4 reports the participants’ anthropometrics and load conditions in each trial. Participants brought to school on average 15% of their body mass (95CI: P1: 13.68–17.31%; P2: 13.30–16.89%; P3: 12.29–15.84%).

### Kinematics

Spatiotemporal gait parameters were monitored by a floor-based photocell system (OptoGait, Mircrogate, Bolzano, Italy) at 1 kHz over 10 m (10 parallel transmitting-receiving bars of 1 m length each, containing 96 LED cells, 3 mm above the ground; hence, accuracy: ~1 cm). When subjects passed between two bars positioned parallel on the ground, transmission and reception would be blocked by their feet. Timing, size, and distance are sensed, being the spatiotemporal parameters then derived^29^. This system was reported as valid to measure spatiotemporal gait^29,30^. Subjects should start and stop walking about 1.5 m before and after the end of the 10 m walkaway, in order to collect data at a steady pace. Data was processed with the software provided by the manufacturer. The average speed, cadence, stride length, durations of the stance, swing and double support phases were assessed. The average values of all steps over the 10 m length were used for further analysis.

### Kinetics

To obtain the gait kinetics, an insole measurement system (Novel Pedar-X, Munich, Germany) was used to collect continuously at 100 Hz the plantar pressure and force distributions. Each insole contains 99 capacitive pressure sensors (2.5 mm thick; spatial resolution of approximately 10 mm; spatial resolution of 1.6 to 2.2 cm^2^; working dynamic range of 15–600 kPa). Data was stored in a data log box that was inside the backpack. Concurrently data was transferred by Bluetooth to a laptop. Biosignal was processed on Pedar®-X software. A calibration device (Novel Trublu, Germany) was used to calibrate the insoles’ sensors before data collection, referring to the manufacturer’s guidelines. The reliability of this system was reported elsewhere^31,32^. The insoles were placed inside the participants’ shoes. On contrary of pressure mats, insoles enable to collect several foot strikes in a relatively short distance and in only a few trials. Another advantage is the possibility of collecting concurrently data from both feet. Moreover, there is no significant change in the gait cycle as happens using pressure mats due to subjects targeting the centre of a relatively small area. Time-series of each trial were displayed on the laptop screen, visually inspected to confirm the quality of the biosignal acquired. If needed, subject would be requested to perform the trial once again. It was extracted the mean pressure, contact area, pressure-time integral and force-time integral under four regions of the feet (toes, metatarsus, midfoot and heel). All variables were calculated as mean value of left and right feet (no significant differences were found in the selected variables between both feet).

### Statistical analysis

Data was described as mean ± 1 SD. Mixed ANOVAs (between- x within-subject analysis; 3 school levels x 4 loads; p ≤ 0.05) was performed for each selected variable. All assumptions to run ANOVAs have been checked beforehand, including normality and sphericity. Degrees of freedom were corrected whenever sphericity’s assumption was violated. If needed, analysis of the variations and mean differences between conditions were carried out by one-way or repeated-measures ANOVAs followed-up by Bonferroni post-hoc test (p ≤ 0.05).

Effect size was computed by eta-squared (ƞ²) and deemed as: without effect if 0 < ƞ² ≤ 0.04; minimum if 0.04 < ƞ² ≤ 0.25; moderate if 0.25 < ƞ² ≤ 0.64 and; strong if ƞ² > 0.64. Whenever suitable and appropriate, Cohen’s d was also computed: (i) small effect size 0 ≤ |d| ≤ 0.2; (ii) medium effect size if 0.2 < |d| ≤ 0.5 and; (iii) large effect size if |d| > 0.5.

To compute the sample power, it was assumed as inputs an expected medium/moderate effect size (for instance, f = 0.25), 5% of error probability for 95% of power, three groups (i.e., 3 school levels), four measurements (i.e., 4 loading conditions), correlation among repeated measures of 0.5 and nonsphericity correction of 1. These inputs yielded a sample size of at least 45 participants (15 in each school level).

## Data Availability

The datasets generated and/or analysed during the current study are available from the corresponding author on reasonable request.
